# Genome-wide SNP discovery and evaluation of genetic diversity among six Chinese indigenous cattle breeds in Sichuan

**DOI:** 10.1371/journal.pone.0201534

**Published:** 2018-08-08

**Authors:** Wei Wang, Jia Gan, Donghui Fang, Hui Tang, Huai Wang, Jun Yi, Maozhong Fu

**Affiliations:** Animal Breeding and Genetics Key Laboratory of Sichuan Province, Sichuan Animal Science Academy, Chengdu, China; Banaras Hindu University, INDIA

## Abstract

Indigenous cattle in Sichuan Province, southwestern China, provide abundant genetic resources. However, their genetic diversity and population structure remain largely unknown, especially on the genome-wide scale. In the present study, we successfully employed the restriction site-associated DNA sequencing approach (RADseq) to explore genome-wide SNPs among six breeds of Sichuan cattle. A total of 238,725 high-confidence SNPs were finally obtained with a mean distance of 11,140 bp between two adjacent sites, and 43.4% were revealed to be novel in comparison with a public reference database of genetic variants. The mean nucleotide diversity and polymorphism information content (PIC) among all six breeds were 0.1878 and 0.1555, respectively. Pingwu and Ganzi cattle showed the highest and lowest genetic diversity, respectively. The inter-breed comparisons revealed that Ganzi and Ebian cattle were obviously separate from the others. Our reference set of genome-wide SNPs specific to indigenous cattle in Sichuan is the first of its kind. Moreover, our set can be used to investigate the genetic diversity and population structure and for genome-wide association studies.

## Introduction

As one of the world’s earliest domesticated mammals[1], modern cattle (*Bos taurus*) have played an important role in livestock husbandry by providing a large amount of milk, meat, hides and other products. Since the initial domestication in the Neolithic age, cattle have been widely disseminated along with human migrations and have adaptively developed considerable variation in appearance and performance[2]. More than 3,200 cattle breeds worldwide have been registered in the Domestic Animal Diversity Information System (DAD-IS) of FAO (http://dad.fao.org/), which systematically reviews historic and current breed classifications[3]. However, only a few dozen cattle breeds, such as Holstein, Angus, Simmental and Hereford, are prevalent throughout the world.

Chinese indigenous cattle were first imported into Northern China as taurine cattle (*B*. *taurus*) between 3000 and 2000 BC, followed by the migration of zebu cattle (*B*. *indicus*) into Southern China via the northwestern route[4]. Therefore, all Chinese indigenous cattle can be obviously classified as humpless breeds distributed in Northern China, humped breeds in Southern China, and mixed populations in middle China according to phenotypic characteristics[5] as well as molecular evidence based on nuclear and mitochondrial DNA[6–8]. At least 53 indigenous cattle breeds were officially recognized based on the national survey of genetic resources in 2010[9]. These genetic resources are expected to substantially contribute to the sustainable development of cattle husbandry in China.

Genome-wide studies focused on population genetics, phylogeography and conservation biology have been greatly facilitated by rapid advances in high-throughput sequencing technologies[10]. In recent years, the restriction site-associated DNA sequencing approach (RADseq) has received increasing attention based on its ability to efficiently identify genome-wide variations at relatively low cost[11]. The technical strengths and weaknesses of RADseq have been recently comprehensively reviewed in applications in ecological and evolutionary genomics[12]. Similar to RADseq, other technologies utilize restriction enzymes to produce a reduced representation of the genome for high-throughput sequencing, such as the reduced representation libraries (RRLs)[13] and genotyping-by-sequencing (GBS) approaches[14]. Using deep sequencing of RRLs, genome-wide single-nucleotide polymorphisms (SNPs) were explored and used to study the population structure of three cattle populations[13]. Subsequently, the GBS approach was successfully applied for SNPs discovery and genotyping in seven taurine and zebu breeds[15].

In the present study, we employed the RADseq approach for *de novo* discovery and genotyping of genome-wide SNPs among six indigenous cattle breeds that are widely distributed in Sichuan Province, China. The results could significantly improve our understanding of the genetic diversity of these indigenous breeds and provide a comprehensive set of candidate genetic markers that are applicable in the association analysis and genetic mapping of economically important traits in Chinese cattle.

## Materials and methods

### Ethics statement

Principles of laboratory animal care were followed, and all procedures were conducted according to the guidelines established by the National Institutes of Health. Every effort was made to minimize suffering. This study was approved by the Animal Experiment Committee of Sichuan Animal Science Academy. All blood samples were collected by local veterinarians for annual health inspections.

### Sample collection and preparation of genomic DNA

**Fig 1:**
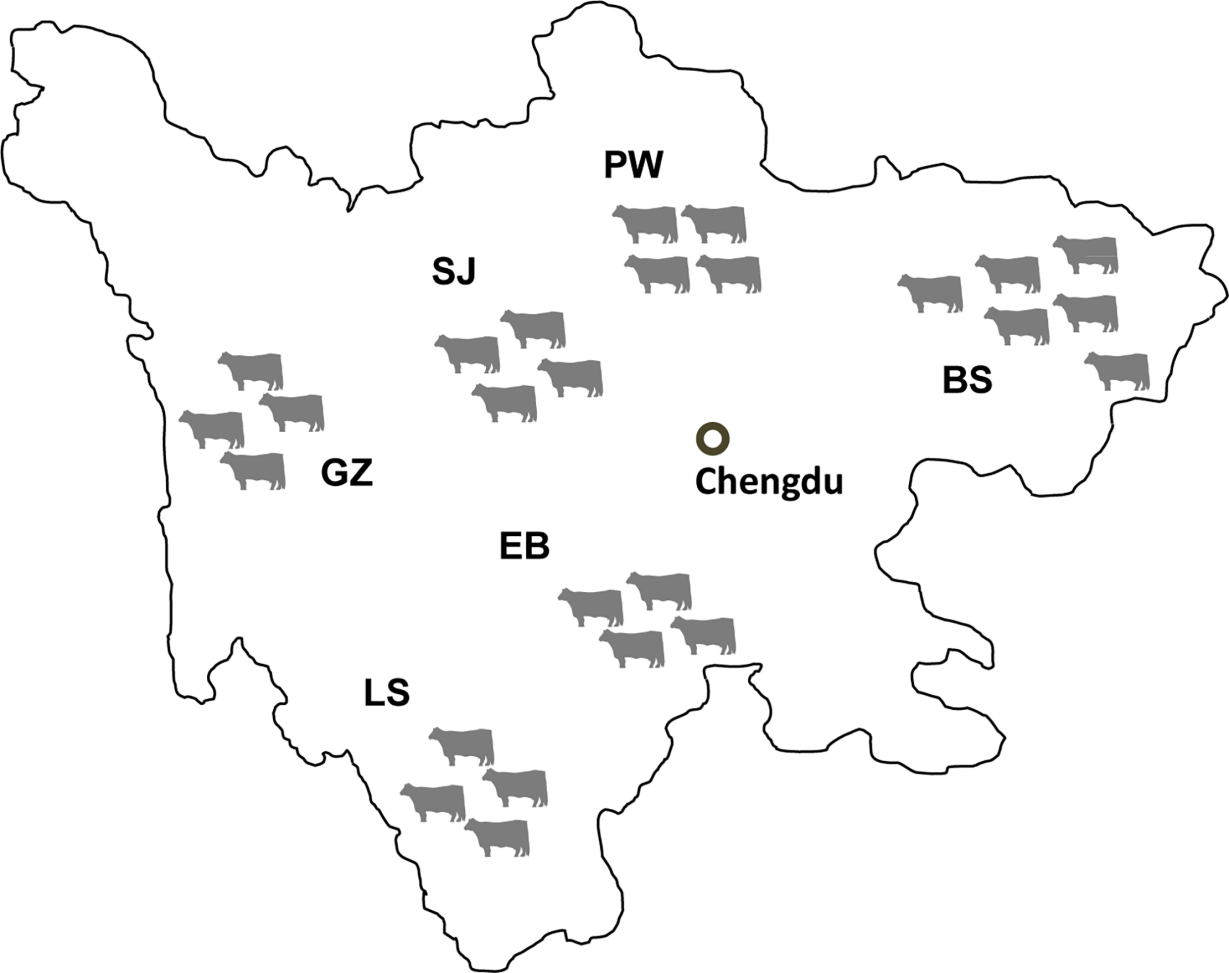
Geographic illustration of the six indigenous cattle breeds collected in Sichuan Province, China. The two-letter abbreviations for every breed are defined in the main text. Please note that this map was created by the authors and is intended for illustrative purposes only.

Blood samples were randomly collected from 55 unrelated animals of six indigenous cattle breeds in Sichuan Province (Fig 1), including 10 Bashan (BS) in Xuanhan County, 8 Pingwu (PW) in Pingwu County, 9 Sanjiang (SJ) in Sanjiang County, 10 Ganzi (GZ) in Daofu County, 9 Liangshan (LS) in Jinyang County and 9 Ebian (EB) cattle in Ebian County. All animals were recruited from rural farmers and did not have genetic relationships with the reference animal, which guaranteed that our samples were as representative as possible. In addition, all included animals had the standard characteristics in appearance according to the classic description[9]. Genomic DNA was extracted using an Axy-Prep Genomic DNA Miniprep Kit (Axygen Bioscience, USA).

### RAD sequencing

Based on a preliminary *in silico* analysis of the cattle genome sequence (UMD3.1), the restriction enzyme *EcoRI* (NEB, Beijing) was successfully used to digest the genomic DNA (~1 μg per sample). We then constructed an RAD sequencing library according to the recommended pipeline[11]. Briefly, the P1 Adapter sequence was first added to the digested fragments, followed by the sequential steps of sample pooling, random shearing, and size selection. The DNA was then ligated to a second adapter (P2) with divergent ends, and fragments of 200 bp to 400 bp and 400 bp to 600 bp were collected for library construction. Finally, the constructed libraries were sequenced on the Illumina HiSeq platform, and 150-bp paired-end reads were generated (Novogene Co. Ltd., Beijing).

### Quality filtering and SNP calling

After the initial sequencing images were converted into sequence files in FASTQ format according to the official pipeline, we investigated the Q_phred_ value-based error rate and GC content along reads using the NGS QC Toolkit[16]. Subsequently, we conducted quality filtering and discarded the low-quality reads, which could be categorized into one of the following types: (i) reads containing adaptor sequences, (ii) reads containing unambiguous bases of N representing more than 10% of the total length, and (iii) reads containing low-quality bases (Q <5) representing more than 50% of the total length. If any member of the paired reads was marked as low quality, both pairs were discarded. After these steps, we obtained clean reads for the following analyses.

All clean reads were mapped against the cattle reference genome (UMD3.1, including chromosomes and all unmapped contigs) using the BWA mapper with default parameters[17]. Subsequently, we employed the GATK toolkit[18] for SNP discovery and genotyping across all 55 samples simultaneously according to the GATK Best Practices recommendations[19, 20]. Duplicate removal, InDel realignment and hard filtering algorithms were performed with default parameters. After obtaining the raw SNPs, we extracted all biallelic SNPs with call rates of 100% (no missing value in any sample) and finally subjected them to the following analyses.

### Data analysis

The coverage depth of reads, nucleotide diversity and test of Hardy-Weinberg equilibrium (HWE) for each locus and the pairwise Identity-by-State (IBS) distances among all cattle were calculated using vcftools[21]. The PopSc toolkit[22] was employed to calculate the polymorphism information content (PIC), Wright’s F_ST_among breeds and Wright’s F_IS_within each breed[23]. The SNP annotations were performed by custom scripts in Python.

## Results

### SNP calling and distribution

**Fig 2:**
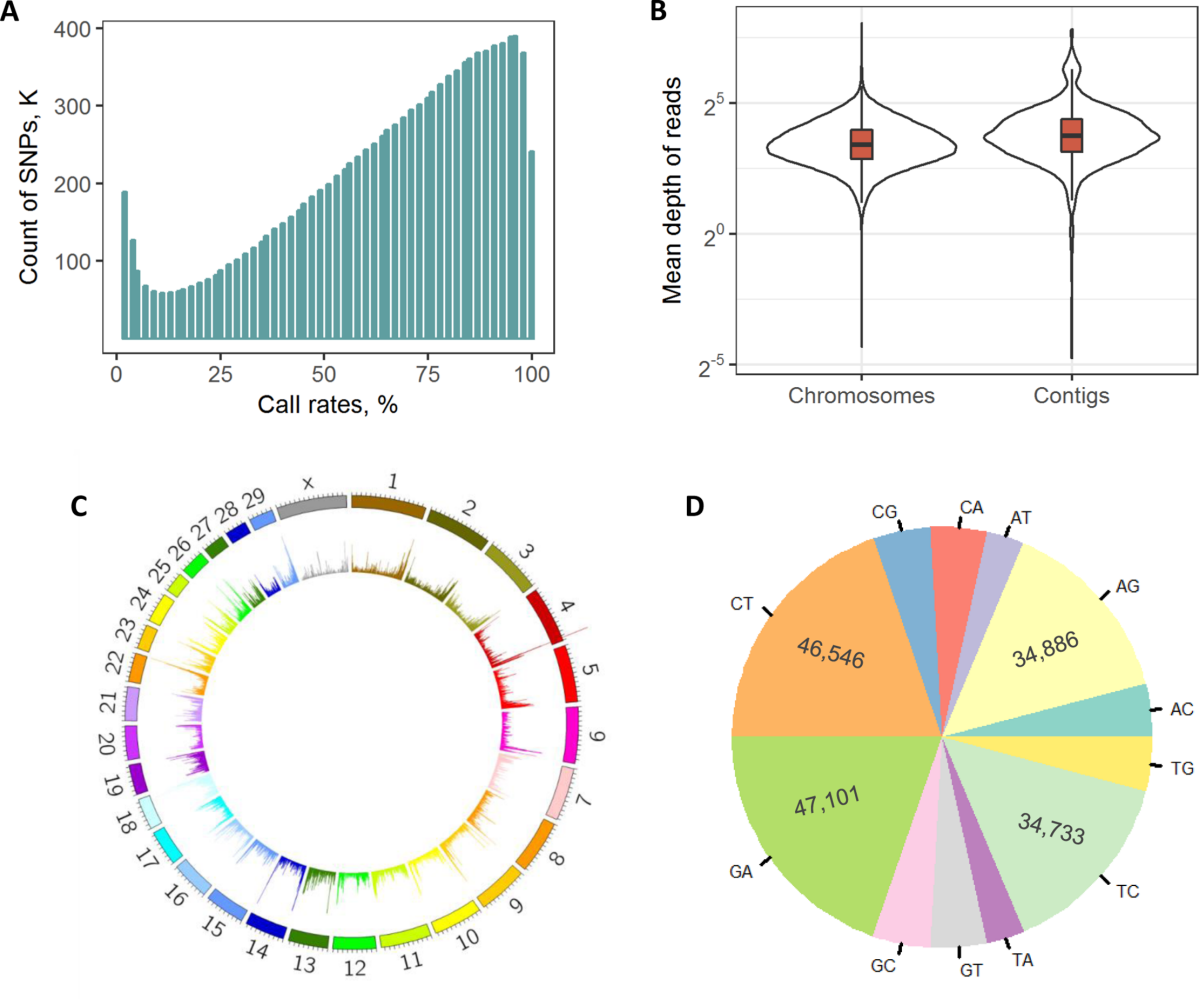
SNP calling and statistical metrics. The call rates of SNPs (A) were first revealed among 55 samples after our hard filtering. The per-site coverage depth of reads was compared among SNPs derived from chromosomes and unanchored contigs (B). The density distributions among 30 chromosomes (C) and substitution types (D) are illustrated. The bin size in (C) was set to 0.2 M for counting SNPs.

We obtained a total of 696 million raw paired-end reads among all samples, which resulted in 692 million clean reads (99.5%) after quality filtering (S1 Table). An average of 98.5% of clean reads were successfully mapped to the reference genome, and 99.1% of them were primary alignments. After performing the local realignment around InDels, a total of 12,043,701 raw SNPs were detected. We first discarded 531,515 low-quality SNPs according to our filtering criterion. Subsequently, the call rates of SNPs among 55 samples were analyzed (Fig 2A), among which only 240,924 SNPs with a call rate of 100% were included in the following analyses. To guarantee reliability, 2,199 non-biallelic alleles were also excluded to produce a clean set consisting of 238,725 SNPs.

All clean SNPs were classified to be derived from these well assembled chromosomes (n = 237,079) and unanchored contigs (1,646), both of them showed similar coverage depths of the sequencing reads (Fig 2B). Furthermore, these clean SNPs were uniformly distributed among all 29 autosomes and the X chromosome, with the highest and lowest counts in chromosomes 11 (N = 10,809) and 28 (N = 4,110), respectively (Fig 2C). The mean distance between two adjacent SNPs was 11,140 bp (S1 Fig). There were 163,266 transitions and 75,459 transversions, leading to an overall transition/transversion ratio of 2.16 (Fig 2D).

### SNP annotation and diversity

**Fig 3:**
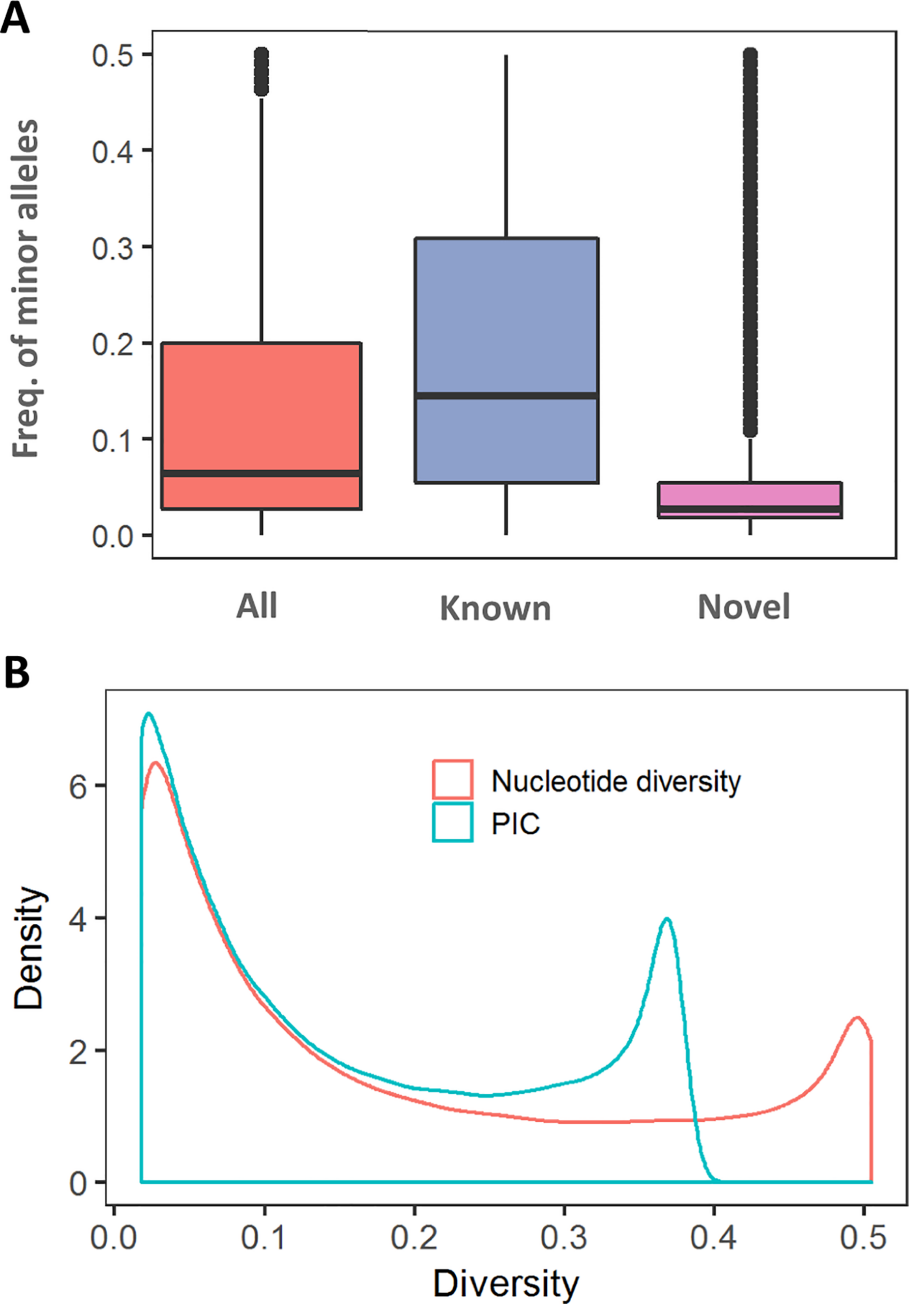
Allelic diversity. The frequencies of minor alleles for known and novel SNPs (A). Density distribution of nucleotide diversity (π) and polymorphism information content (PIC) for all SNPs (B).

By comparison with the reference database of variants available in Ensembl (release 90), all clean SNPs discovered in the present study could be classified into 135,151 known (56.6%) and 103,574 novel SNPs (43.4%). Furthermore, 237,489 SNPs were located within exon (2,578), intron (75,029) and intergenic (159,882) regions. There were 34,002 SNPs (14.2%) with minor allele frequencies lower than 0.01, most of which (N = 25,618) were the novel SNPs discovered in the present study (Fig 3A). Among all SNPs, the distribution density of nucleotide diversity had two peaks at approximately 0.02 and 0.49 (Fig 3B), and a similar pattern was observed for the PIC. The median and mean values of nucleotide diversity were 0.1203 and 0.1878 among all breeds (Table 1). Pingwu cattle showed the highest nucleotide diversity, with a median of 0.3250 and mean of 0.3132. Pingwu cattle also had the highest PIC value among the six breeds of Sichuan cattle.

**Table 1:** Overview of genetic diversity within each breed.

Breeds	Nucleotide diversity		Polymorphism information content	
	Median	Mean	Median	Mean
**Bashan (BS)**	0.2684	0.2821	0.2225	0.2211
**Pingwu (PW)**	0.3250	0.3132	0.2583	0.2408
**Sanjiang (SJ)**	0.2941	0.3055	0.2392	0.2366
**Ganzi (GZ)**	0.1895	0.2665	0.1638	0.2105
**Liangshan (LS)**	0.2491	0.2966	0.2392	0.2302
**Ebian (EB)**	0.2491	0.2995	0.2322	0.2392
Total	**0.1203**	**0.1878**	**0.1121**	**0.1555**

### Population structure

**Fig 4:**
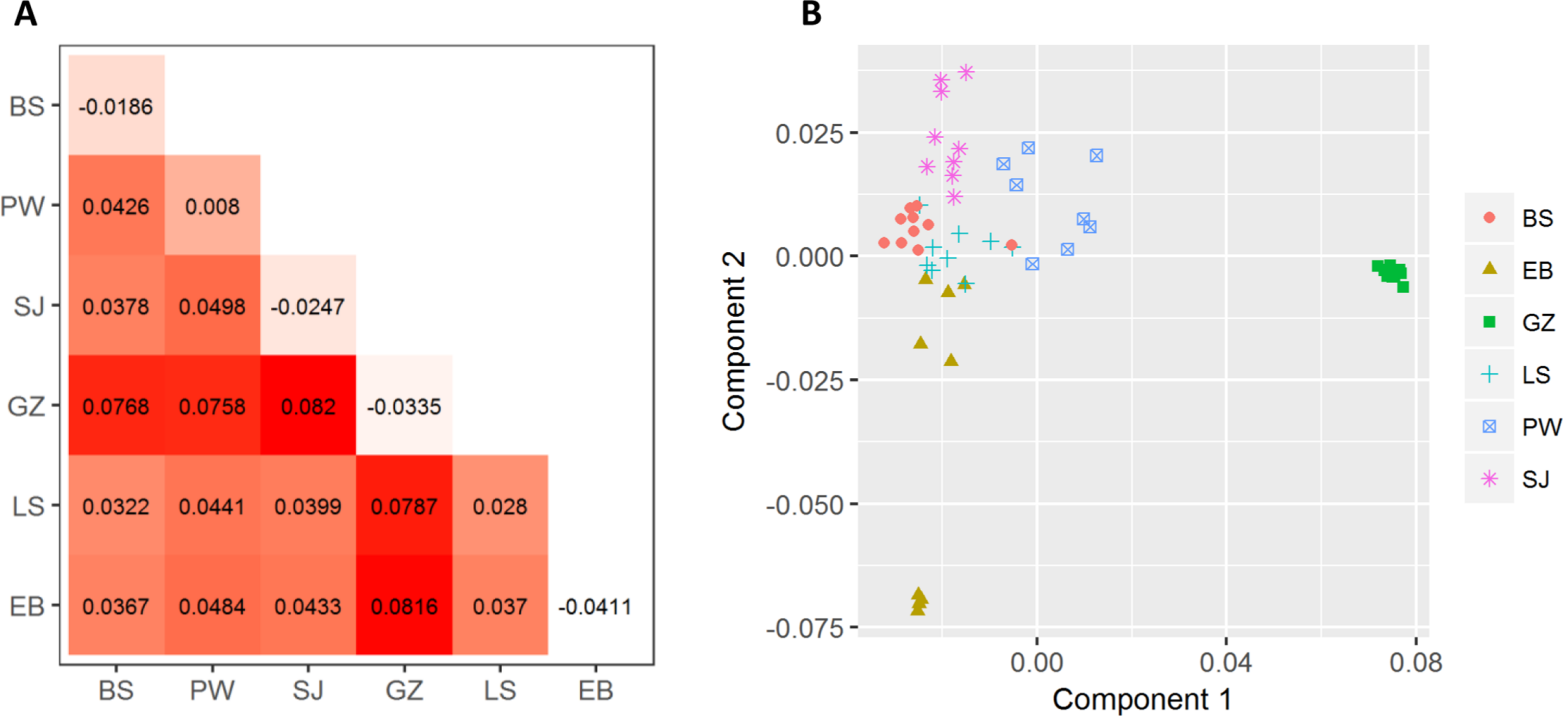
Genetic structure among the six populations. The matrix (A) shows the pairwise Wright’s F_ST_ values in the lower triangular area and F_IS_ values in diagonal cells. The IBS distance-based multidimensional scaling plot of 55 samples is shown in (B).

Among all 55 samples, 197,553 SNPs (82.6%) were revealed to be under HWE with a P threshold value of 0.05. The highest and lowest inter-population differences were observed between Sanjiang and Ganzi cattle (F_ST_ = 0.082) and between Bashan and Liangshan cattle (F_ST_ = 0.032), respectively (Fig 4A). The intra-population inbreeding coefficients (F_IS_) ranged from -0.041 in Ebian cattle to 0.028 in Liangshan cattle. Finally, we calculated the pairwise IBS distances among all samples, which revealed that Ganzi and Ebian cattle were obviously separated from the other breeds (Fig 4B). Within each breed, the inter-individual differences were substantially higher in Sanjiang and Pingwu cattle than in the Ganzi breed. However, the nine Ebian cattle were obviously separated into two subgroups showing intra-breed genetic differentiation.

## Discussion

Sichuan Province is located in southwestern China and shows substantial diversity in geography and climate, resulting in abundant genetic resources for both wild and domesticated animals. Indigenous cattle in Sichuan can be classified into seven breeds according to their geographic distributions and various phenotypic characteristics[9]. The effective conservation and exploration of genetic resources are expected to be important for promoting the sustainable development of cattle husbandry. Although Sichuan cattle have been already included in a few sporadic reports on genetic diversity based on mitochondrial DNA[6, 24] and microsatellite markers[8], a systematic investigation of genetic diversity and population structure requires the inclusion of more breeds and extension to the genome-wide level.

In the present study, we collected six breeds of Sichuan indigenous cattle and obtained a reference set of genome-wide SNPs by high-throughput sequencing technology. Among the 238 thousand SNPs discovered in this study, as many as 43.4% were novel in comparison with the public reference database; this result obviously supports the necessity of *de novo* discovery of genetic variants in Sichuan or even Chinese indigenous cattle due to the large number of breed-specific variant sites. However, more caution should be paid to the novel SNPs with minor allele frequencies of less than 0.01, which are prone to generating false-positive results due to the limited number of samples analyzed in the present study. Upon completing the draft genome sequence of cattle in 2009, a genome-wide SNP panel was successfully constructed and used to reveal the gene structure among 19 geographically and biologically diverse breeds[25]. Although there are approximately 88 million reference SNPs of cattle available in the Ensembl database (release 90), nearly all were obtained in European cattle. Therefore, we believe that the genomic discovery of SNPs in Chinese cattle will significantly expand the public SNP database for cattle.

Among the six breeds of Sichuan cattle included in the present study, we observed obvious differences in the level of genetic diversity. Ganzi cattle are mainly distributed in northwestern Sichuan and show the lowest genetic diversity, whereas the highest genetic diversity is observed in Pingwu cattle distributed in northern Sichuan. Based on 30 microsatellite markers, Zhang and colleagues[8] previously reported PIC values of 0.680 and 0.730 for Sanjiang and Ebian cattle, respectively, both of which are significantly higher than the PIC indices of SNPs calculated in the present study. Unfortunately, we were unable to directly compare the six breeds of Sichuan cattle with other Chinese cattle breeds or with European cattle based on this SNP panel because reference data on allele frequencies are unavailable. According to genetic distance-based clustering, Ganzi and Ebian cattle are obviously separate from other breeds and from each other, which would therefore suggest that there are a total of three different genetic lineages in Sichuan indigenous cattle. In contrast to other breeds, Ebian cattle exhibit a mixed gene pool of two of these genetic lineages. Similarly, our calculated F_ST_ values are also lower than those in a report based on microsatellite markers[8]. We believe that these differences are attributable to the use of different genetic markers and our relatively small sample size.

In conclusion, we successfully explored genome-wide SNP markers among six breeds of Sichuan indigenous cattle by high-throughput sequencing technology. In addition to improving the understanding of the genetic diversity and population structure among these breeds, this set of SNPs will greatly facilitate genetic association analysis of economically important traits in Sichuan cattle.

## Supporting information

S1 Fig **Distances between two adjacent SNPs among the whole set of clean SNPs (A) and for SNPs in each chromosome (B)**.
(DOCX)

S1 Table Sequencing and quality filtering of reads. (DOCX)
