# Supplementary material for: Genome-wide SNP discovery and evaluation of genetic diversity among six Chinese indigenous cattle breeds in Sichuan
Source: PLoS One. 2018 Aug 8;13(8):e0201534. doi: 10.1371/journal.pone.0201534 (PMC6082554; doi:10.1371/journal.pone.0201534)

**Genome-wide SNP discovery and evaluation of genetic diversity among six Chinese indigenouscattle breeds in Sichuan**

Wei Wang^1^, Jia Gan^1^, Donghui Fang^1^, Hui Tang^1^, Huai Wang^1^, Jun Yi^1^*, Maozhong Fu^1^*,

^1^ Animal Breeding and Genetics Key Laboratory of Sichuan Province, Sichuan Animal Science Academy, Chengdu, China.

* Corresponding authors

E-mail: [fmz847464621@163.com](mailto:fmz847464621@163.com) (MF); E-mail: [372197981@QQ.com](mailto:372197981@QQ.com) (JY)

**S1 Fig**. Distances between two adjacent SNPs among the whole set of clean SNPs (A) and for SNPs in each chromosome (B).


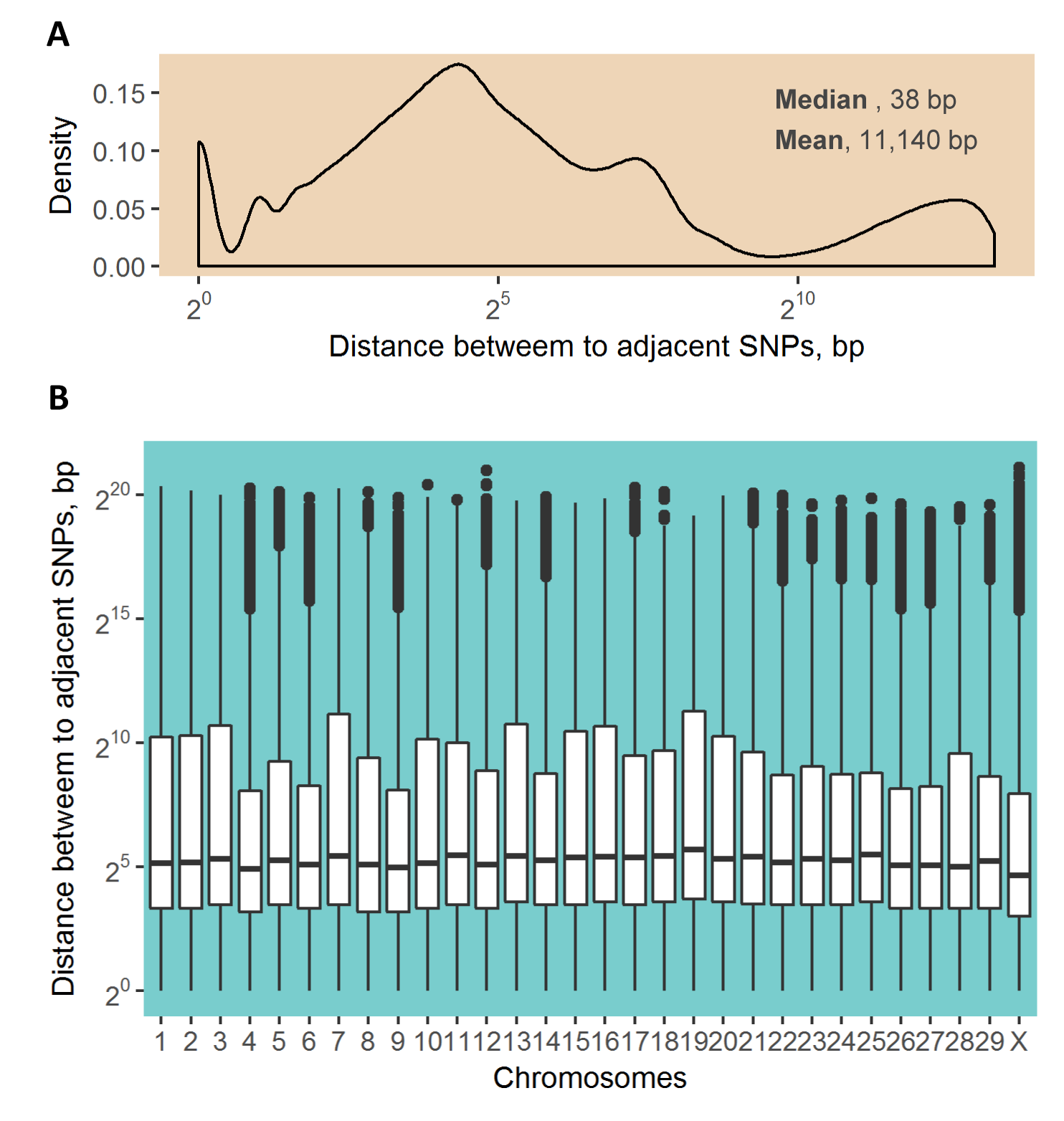

Supplement: S1 Fig — Distances between two adjacent SNPs among the whole set of clean SNPs (A) and for SNPs in each chromosome (B). (DOCX) [file pone.0201534.s001.docx]
