# Supplementary material for: Genome-wide SNP discovery and evaluation of genetic diversity among six Chinese indigenous cattle breeds in Sichuan
Source: PLoS One. 2018 Aug 8;13(8):e0201534. doi: 10.1371/journal.pone.0201534 (PMC6082554; doi:10.1371/journal.pone.0201534)
Supplement: S1 Table — (DOCX) [file pone.0201534.s002.docx]

**Genome-wide SNP discovery and evaluation of genetic diversity among six Chinese indigenouscattle breeds in Sichuan**

Wei Wang^1^, Jia Gan^1^, Donghui Fang^1^, Hui Tang^1^, Huai Wang^1^, Jun Yi^1^*, Maozhong Fu^1^*,

^1^ Animal Breeding and Genetics Key Laboratory of Sichuan Province, Sichuan Animal Science Academy, Chengdu, China.

* Corresponding authors

E-mail: [fmz847464621@163.com](mailto:fmz847464621@163.com) (MF); E-mail: [372197981@QQ.com](mailto:372197981@QQ.com) (JY)

**S1 Table.** Sequencing and quality filtering of reads

| SampleID | Raw reads | Clean reads | Raw bases(G) | Clean bases(G) | Effective rate(%) | Error rate(%) | Q20(%) | Q30(%) | GC content(%) |
| --- | --- | --- | --- | --- | --- | --- | --- | --- | --- |
| EB10 | 12,436,598 | 12,402,548 | 3.731 | 3.721 | 99.73 | 0.03 | 96.33 | 91.64 | 43.75 |
| EB19 | 9,914,142 | 9,889,188 | 2.974 | 2.967 | 99.75 | 0.03 | 95.48 | 90 | 45.19 |
| EB2 | 10,725,141 | 10,698,245 | 3.218 | 3.209 | 99.75 | 0.03 | 94.92 | 89.07 | 46.25 |
| EB22 | 14,063,421 | 14,039,079 | 4.219 | 4.212 | 99.83 | 0.03 | 96.94 | 92.56 | 45.28 |
| EB24 | 13,872,234 | 13,249,377 | 4.162 | 3.975 | 95.51 | 0.02 | 97.62 | 94.33 | 46.3 |
| EB3 | 10,601,530 | 10,578,525 | 3.18 | 3.174 | 99.78 | 0.03 | 95.08 | 89.23 | 47.21 |
| EB4 | 12,802,389 | 12,504,118 | 3.841 | 3.751 | 97.67 | 0.03 | 96.26 | 91.64 | 46.67 |
| EB5 | 18,523,425 | 18,474,885 | 5.557 | 5.542 | 99.74 | 0.03 | 97.03 | 92.88 | 42.58 |
| EB8 | 11,015,877 | 10,983,206 | 3.305 | 3.295 | 99.7 | 0.03 | 95.87 | 90.62 | 45.88 |
| GZ11 | 11,987,001 | 11,947,697 | 3.596 | 3.584 | 99.67 | 0.04 | 94.11 | 87.35 | 47.3 |
| GZ13 | 13,420,135 | 13,362,450 | 4.026 | 4.009 | 99.57 | 0.03 | 96.04 | 90.94 | 43.52 |
| GZ15 | 11,147,860 | 11,090,650 | 3.344 | 3.327 | 99.49 | 0.03 | 95.53 | 89.96 | 47.52 |
| GZ19 | 14,970,468 | 14,903,322 | 4.491 | 4.471 | 99.55 | 0.03 | 95.99 | 90.9 | 48.11 |
| GZ2 | 10,878,044 | 10,844,672 | 3.263 | 3.253 | 99.69 | 0.03 | 95.72 | 90.27 | 48.86 |
| GZ20 | 16,355,393 | 16,309,436 | 4.907 | 4.893 | 99.72 | 0.03 | 96.5 | 91.74 | 46.75 |
| GZ3 | 12,992,276 | 12,963,553 | 3.898 | 3.889 | 99.78 | 0.03 | 96.58 | 91.84 | 49.61 |
| GZ4 | 13,697,478 | 13,639,307 | 4.109 | 4.092 | 99.58 | 0.02 | 97.48 | 93.75 | 45.69 |
| GZ7 | 11,039,605 | 10,969,930 | 3.312 | 3.291 | 99.37 | 0.03 | 95.65 | 90.23 | 46.77 |
| GZ8 | 13,575,943 | 13,529,270 | 4.073 | 4.059 | 99.66 | 0.03 | 97.07 | 92.94 | 46.04 |
| LS13 | 15,384,255 | 15,313,589 | 4.615 | 4.594 | 99.54 | 0.03 | 96.83 | 92.06 | 46.16 |
| LS14 | 9,764,379 | 9,577,885 | 2.929 | 2.873 | 98.09 | 0.03 | 95.31 | 89.8 | 44.5 |
| LS15 | 11,876,112 | 11,806,719 | 3.563 | 3.542 | 99.42 | 0.04 | 93.69 | 87.41 | 42.84 |
| LS18 | 9,727,103 | 9,677,614 | 2.918 | 2.903 | 99.49 | 0.03 | 95.48 | 90.12 | 43.82 |
| LS2 | 15,121,646 | 15,032,056 | 4.536 | 4.51 | 99.41 | 0.03 | 96.66 | 91.81 | 43.03 |
| LS5 | 14,425,782 | 14,370,060 | 4.328 | 4.311 | 99.61 | 0.03 | 96.39 | 91.16 | 46.16 |
| LS6 | 13,252,496 | 13,199,871 | 3.976 | 3.96 | 99.6 | 0.03 | 96.79 | 92.03 | 47.96 |
| LS8 | 13,186,079 | 13,125,127 | 3.956 | 3.938 | 99.54 | 0.03 | 96.33 | 91.41 | 43.97 |
| LS9 | 11,347,176 | 11,238,143 | 3.404 | 3.371 | 99.04 | 0.02 | 97.81 | 94.36 | 43.41 |
| PW13 | 11,701,683 | 11,670,264 | 3.511 | 3.501 | 99.73 | 0.03 | 94.5 | 88.7 | 46.52 |
| PW15 | 13,999,447 | 13,922,262 | 4.2 | 4.177 | 99.45 | 0.03 | 95.77 | 90.33 | 45.3 |
| PW16 | 11,777,759 | 11,742,954 | 3.533 | 3.523 | 99.7 | 0.03 | 95.77 | 90.4 | 47.94 |
| PW27 | 11,684,708 | 11,622,185 | 3.505 | 3.487 | 99.46 | 0.03 | 96.99 | 93.14 | 45.16 |
| PW30 | 13,438,556 | 13,378,312 | 4.032 | 4.013 | 99.55 | 0.03 | 97.2 | 93.45 | 44.44 |
| PW4 | 11,394,311 | 11,335,663 | 3.418 | 3.401 | 99.49 | 0.03 | 96.32 | 91.5 | 43.92 |
| PW5 | 13,726,845 | 13,689,012 | 4.118 | 4.107 | 99.72 | 0.02 | 97.41 | 93.47 | 44.81 |
| PW7 | 12,108,712 | 12,075,751 | 3.633 | 3.623 | 99.73 | 0.03 | 94.59 | 88.94 | 44.3 |
| SJ17 | 15,409,090 | 15,345,094 | 4.623 | 4.604 | 99.58 | 0.03 | 97.32 | 93.66 | 43.25 |
| SJ18 | 11,025,152 | 10,984,548 | 3.308 | 3.295 | 99.63 | 0.03 | 96.17 | 91.05 | 49.04 |
| SJ19 | 10,324,200 | 10,291,275 | 3.097 | 3.087 | 99.68 | 0.03 | 96.1 | 90.87 | 49.19 |
| SJ22 | 10,900,852 | 10,868,250 | 3.27 | 3.26 | 99.7 | 0.03 | 95.86 | 90.65 | 45.12 |
| SJ24 | 13,100,086 | 13,045,625 | 3.93 | 3.914 | 99.58 | 0.03 | 96.48 | 92.1 | 45.09 |
| SJ3 | 16,239,594 | 16,076,197 | 4.872 | 4.823 | 98.99 | 0.03 | 95.98 | 90.95 | 53.44 |
| SJ4 | 10,331,938 | 10,297,282 | 3.1 | 3.089 | 99.66 | 0.03 | 96.94 | 92.94 | 46.07 |
| SJ6 | 11,291,751 | 11,254,528 | 3.388 | 3.376 | 99.67 | 0.03 | 95.59 | 89.93 | 44.43 |
| SJ8 | 11,394,308 | 11,355,837 | 3.418 | 3.407 | 99.66 | 0.03 | 96.16 | 91.23 | 45.95 |
| BS1 | 10,781,267 | 10,758,491 | 3.234 | 3.228 | 99.79 | 0.03 | 96.32 | 91.06 | 45.6 |
| BS10 | 13,959,373 | 13,900,089 | 4.188 | 4.17 | 99.58 | 0.03 | 96.56 | 91.99 | 46.08 |
| BS11 | 16,249,478 | 16,204,590 | 4.875 | 4.861 | 99.72 | 0.03 | 97.11 | 93.04 | 45.44 |
| BS12 | 13,114,840 | 13,056,234 | 3.934 | 3.917 | 99.55 | 0.04 | 93.78 | 87.44 | 45.64 |
| BS14 | 10,740,212 | 10,689,119 | 3.222 | 3.207 | 99.52 | 0.03 | 95.1 | 89.15 | 49.05 |
| BS21 | 12,023,519 | 11,981,946 | 3.607 | 3.595 | 99.65 | 0.03 | 94.64 | 88.98 | 43.96 |
| BS22 | 10,833,866 | 10,799,194 | 3.25 | 3.24 | 99.68 | 0.03 | 96 | 90.79 | 45.85 |
| BS24 | 13,545,179 | 13,488,166 | 4.064 | 4.046 | 99.58 | 0.03 | 96.91 | 92.94 | 43.32 |
| BS3 | 15,682,573 | 15,601,278 | 4.705 | 4.68 | 99.48 | 0.02 | 97.63 | 94.27 | 43.36 |
| BS98 | 11,160,533 | 11,125,922 | 3.348 | 3.338 | 99.69 | 0.03 | 95.53 | 89.69 | 46.59 |
| Mean | **12,655,342** | **12,586,920** | **3.797** | **3.776** | **99.46** | **0.03** | **96.11** | **91.18** | **45.82** |
